# Prevalence of extended-spectrum betalactamase producing–Enterobacteriaceae (ESBL-E) carriage on admission at Geneva University Hospitals (HUG)

**DOI:** 10.1186/2047-2994-4-S1-P120

**Published:** 2015-06-16

**Authors:** C Fankhauser, J Schrenzel, V Prendki, F Ris, E Schiffer, P Gastmeier, Y Carmeli, D Pittet, S Harbarth

**Affiliations:** 1 Infection Control Programme, Geneva University Hospitals, Geneva, Switzerland; 2 Bacteriology Laboratory, Geneva University Hospitals, Geneva, Switzerland; 3 Geriatrics, Internal Medicine and Rehabilitation Service, Geneva University Hospitals, Geneva, Switzerland; 4 Visceral Surgery Service, Geneva University Hospitals, Geneva, Switzerland; 5 Anesthesiology Service, Geneva University Hospitals, Geneva, Switzerland; 6 R-Gnosis WP5 Leader, Charité-University Hospital, Berlin, Germany; 7 R-Gnosis WP4 Leader, Sourasky Medical Center, Tel-Aviv, Israel

## Introduction

The increasing prevalence of ESBL-E in the community is a cause of concern for hospitals. Early detection of ESBL-E carriers on admission could allow timely implementation of control measures or appropriate selection of antimicrobials.

## Objectives

To describe the current prevalence of ESBL-E rates upon admission to 4 different services at HUG, in the context of a multicenter European study (R-Gnosis).

## Methods

Patients admitted to 4 different services were screened by rectal swabs on admission. From January 2014 through January 2015, patients admitted to 4 wards, including: Ortho1 (sport traumatology), Ortho 2 (septic); Geriatrics (2 wards) and patients undergoing elective colorectal surgery (ECS) were screened from April 2013-October 2014.

## Results

Overall, from 2394 admitted patients, 2136 were screened on admission (89.2%). Median age was 67.3 years (SD±20.9); 51.6% were male. Only 92/2136 (4.3%) had a previously known status of ESBL carriage. A total of 226/2136 (10.6%) patients were found to be ESBL-E carriers: *E. coli* (n=166; 73.4%); *K. pneumoniae* (n=26; 11.5%) and other Enterobacteriaceae (n=34; 15.0%). Among *K. pneumonia* carriers on admission, 24/26 (92.3%), had a previous hospitalization less than 12 months before admission screening and 21/26 (80.8%) had the previous hospitalization within 3 months only. ESBL-E carriage was 83/981 (8.4%) and 61/430 (14.2%) for Ortho 1 and 2 respectively; Geriatrics, 42/371 (11.3%); and ECS, 41/354 (11.6%).

## Conclusion

Overall 10.6% of patients screened were ESBL-E carriers upon admission at HUG, mostly due to ESBL-producing *E. coli*. Patients admitted to septic orthopedics, geriatrics and ECS had a higher prevalence on admission. The majority of ESBL- *Klebsiella pneumonia* carriers had a recent history of hospitalization.

## Disclosure of interest

None declared.

